# Serum extracellular vesicles derived hsa-miR-320d as an indicator for progression of clear cell renal cell carcinoma

**DOI:** 10.1007/s12672-023-00730-2

**Published:** 2023-06-28

**Authors:** Yizheng Xue, Tianyi Chen, Naiqiao Hou, Xiaorong Wu, Wen Kong, Jiwei Huang, Jin Zhang, Yonghui Chen, Junhua Zheng, Wei Zhai, Wei Xue

**Affiliations:** 1 Department of Urology, Renji Hospital, Shanghai Jiao Tong University School of Medicine, 160# Pu Jian Ave, Shanghai, 200127 China; 2 State Key Laboratory of Oncogenes and Related Genes, Department of Urology, School of Medicine, Renji Hospital, Shanghai Jiao Tong University, Shanghai, 200127 China

**Keywords:** Extracellular vesicles, Clear cell renal cell carcinoma, Biomarker, Hsa-miR-320d

## Abstract

**Background:**

Renal cell carcinoma (RCC) is a prevalent malignancy with a rising incidence in developing countries. Clear cell renal cell carcinoma (ccRCC) constitutes 70% of RCC cases and is prone to metastasis and recurrence, yet lacks a liquid biomarker for surveillance. Extracellular vesicles (EVs) have shown promise as biomarkers in various malignancies. In this study, we investigated the potential of serum EV-derived miRNAs as a biomarker for ccRCC metastasis and recurrence.

**Materials and methods:**

Patients diagnosed with ccRCC between 2017 and 2020 were recruited in this study. In the discovery phase, high throughput small RNA sequencing was used to analyze RNA extracted from serum EVs derived from localized ccRCC (LccRCC) and advanced ccRCC (AccRCC). In the validation phase, qPCR was employed for quantitative detection of candidate biomarkers. Migration and invasion assays were performed on ccRCC cell line OSRC2.

**Results:**

Serum EVs derived hsa-miR-320d was significantly up-regulated in patients with AccRCC than in patients with LccRCC (p < 0.01). In addition, Serum EVs derived hsa-miR-320d was also significantly up-regulated in patients who experienced recurrence or metastasis (p < 0.01). Besides, hsa-miR-320d enhances the pro-metastatic phenotype of ccRCC cells in vitro.

**Conclusions:**

Serum EVs derived hsa-miR-320d as a liquid biomarker exhibits significant potential for identifying the recurrence or metastasis of ccRCC, as well as hsa-miR-320d promotes ccRCC cells migration and invasion.

**Supplementary Information:**

The online version contains supplementary material available at 10.1007/s12672-023-00730-2.

## Introduction

Renal cell carcinoma(RCC) is a common urinary malignant tumor with an annual increase in incidence in developing country [1, 2]. Clear cell renal cell carcinoma(ccRCC) which originates from the renal proximal tubule [3], constitutes approximately 70% of RCC cases and is associated with a relatively poor prognosis, high recurrence or metastasis rates [4]. Thirty percent of ccRCC patients experienced early relapse after surgery, with few symptomatic [5]. Surveillance of RCC depends on imaging examinations, which may be delayed. Unlike universal lymph node invasion in most urinary malignant tumors [6], distal metastasis is more prevalent in ccRCC than in other types of RCC [7], indicating that metastasis of ccRCC depends on blood flow, especially the premetastatic microenvironment comprised of circulating tumor cells and extracellular vesicles [8]. Thus the potential of liquid biopsy in identifying progressive ccRCC is anticipated.

Acknowledged ccRCC prognostic models such as SSIGN or UISS rely heavily on pathological information, specifically TNM stage, tumor size, WHO/ISUP grade, sarcomatoid differentiation, hemorrhage, or necrosis [9]. Unlike prostate cancer, ccRCC lacks a widely recognized liquid biomarker that can aid in non-invasive testing and provide inference for treatment and surveillance [10]. Therefore, it is important to identify liquid biomarkers for ccRCC hazard stratification.

Extracellular vesicles(EVs) are circulating nano vesicles derived from cells, composed of nucleic acids, proteins, and metabolites within a lipid bilayer membrane that stabilizes these contents [11], which is now widely used as a reliable tool for cancer diagnosis [12, 13]. EVs are responsible for intercellular communication and play a crucial role in the formation of the premetastatic niche [14, 15]. Numerous studies have demonstrated the significant contribution of EVs to the progression of ccRCC [16]. For instance, EVs derived lncARSR transmitted between ccRCC cells to facilitate sunitinib resistance [17], EVs are packaged with lncHILAR under hypoxia to promote ccRCC invasion under normoxia [18], and cancer stem cell(CSC) derived EVs promote EMT of ccRCC [19].

Several studies have taken EVs derived miRNAs as biomarker of ccRCC, identified that serum extracellular vesicles derived miR-210 [20], miR-1233 [21], miR-224 [22] can distinguish ccRCC patients from healthy people, as well as plasma derived miR-let-7i,miR-26a,miR-615 can to some extent distinguish favor risk or poor risk in RCC [23].Urine EVs derived miR-126-3p was also reported as diagnostic biomarker of ccRCC [24]. However, most of these studies used previously published molecules as subjects and validated them in a relatively small cohort without follow up information. In contrast, our study employed a high throughput screening approach to determine whether serum EVs derived hsa-miR-320d is a potential biomarker which is highly expressed in advanced ccRCC, and this conclusion was validated in two seperate cohorts.

## Methods

### Ethical considerations

This study was conducted according to the guidelines of the Declaration of Helsinki and approved by the Shanghai Jiaotong University School of Medicine, Renji Hospital Ethics Committee. Serum samples were obtained from Renji Hospital Biobank, Shanghai Jiaotong University School of Medicine. All the donors signed informed consent forms.

### Serum samples collection

Fasting venous blood samples were collected in non-anticoagulant vacutainers one day before surgery. After collection, each sample was centrifugated at 2000 g for 10 min to extract the serum. All serum samples were stored at − 80 °C in a tube before use.

### EVs isolation and characterization

Total exosome isolation reagent(Invitrogen 4478360) and exoRNeasy Serum Midi Kit(Qiagen 77144) was used to extract EVs from previously thawed serum in discovery cohort and validation cohort 1, respectively. In brief, 170µL total exosome isolation reagent was added to 850µL serum, the mixture was incubated on the ice for 30 min and centrifuged at 10000 g for 10 min, the supernatant was discarded and EVs were deposited. Using exoRNeasy Serum Midi Kit, 400µL XBP was added to 400µL serum and the mixture was added onto the spin column to bind EVs by centrifugation. XWP buffer was used to remove the residual buffer. EVs can be washed out by XE buffer or directly switch to miRNA extraction. For validation cohort 2, EVs were extracted from 200µL serum.

### Small RNA deep sequencing

Extract the total RNA of the EVs, connect the 3'end and 5'linker successively and reverse transcribed into cDNA which carried out PCR amplification. The gel was cut to recover the target fragment library, and the qualified library was sequenced on the Ribobio sever. The clean reads obtained by sequencing were compared to the miRDeep2 and miRBase.v22 databases of all miRNA mature bodies, and their expression was calculated. miRNA expression correction method: Calculate the number of reads aligned to a specific miRNA per million clean reads (the number of reads per million (RPM) clean tags), and the specific calculation formula is as follows:

1 $$RPM\,=\,\frac{number\,of\,reads\,mapping\,to\,miRNA}{number\,of\,reads\,in\,Clean\,data}\times {10}^{6}$$

### Total RNA extraction

700ul QIAzol was added to EVs or EVs-bound membranes. 4 µL cel-miR-40 (10 pmol) was added to the lysate as a spike-in control. 90 µL chloroform was mixed with the lysate and the mixture was centrifuged for 15 min at 12000*g* at 4 °C. 270 µL upper aqueous phase was mixed with 540 ul 100% ethanol and passed through an RNeasy MinElute spin column to bind RNA. The residual buffer was washed out by RWT and RPE, and RNA was eluted with 16 µL RNase free water. For validation cohort 2, 2µL cel-miR-39(20 pmol) was added to EVs lysate as a spike-in control.

### miRNA quantification

TaqMan Small RNA Assays (ThermoFisher 4364031 and 4427975) was used to perform reverse transcription of 5ul total RNA according to previously described procedures. The cDNA, TaKaRa Premix (RR390) and the ABI Step one Plus 96-well PCR system were used in the qRT-PCR assay. The delta Ct method was used for data analysis.

### Statistical analysis

Data were analyzed and presented using GraphPad Prism 8.0 software, shown as the form of median with 95% CI. An unpaired t test was used to analyze the differences between two groups. A 2-tailed P value less than 0.05 indicates a significant difference.

### Wound-healing assay and invasion assay

To investigate migration, OSRC2 cells was cultured in a six-well plate with serum free RMPI 1640. Streaks across the plate were created with a pipette tip and progression of migration was observed and photographed at the beginning and 24 h after wounding. The data shown were representative micrographs of wound-healing assay. The invasive capability of RCC cells was determined by the transwell assay. The membrane was coated with matrigel (Thermofisher). OSRC2 cells was harvested and seeded with serum-free RMPI 1640 into the upper chambers at 2*10^4^ cells/well, and the bottom chambers contained RMPI 1640 with 10% FBS, and then incubated for 24 h at 37 °C. After incubation, the invasive cells that penetrated into the lower surface of the membrane were fixed by 4% paraformaldehyde and stained with 0.5% crystal violet. Experiments were repeated at least three times with consistent results.

## Results

### Study design

**Fig. 1:**
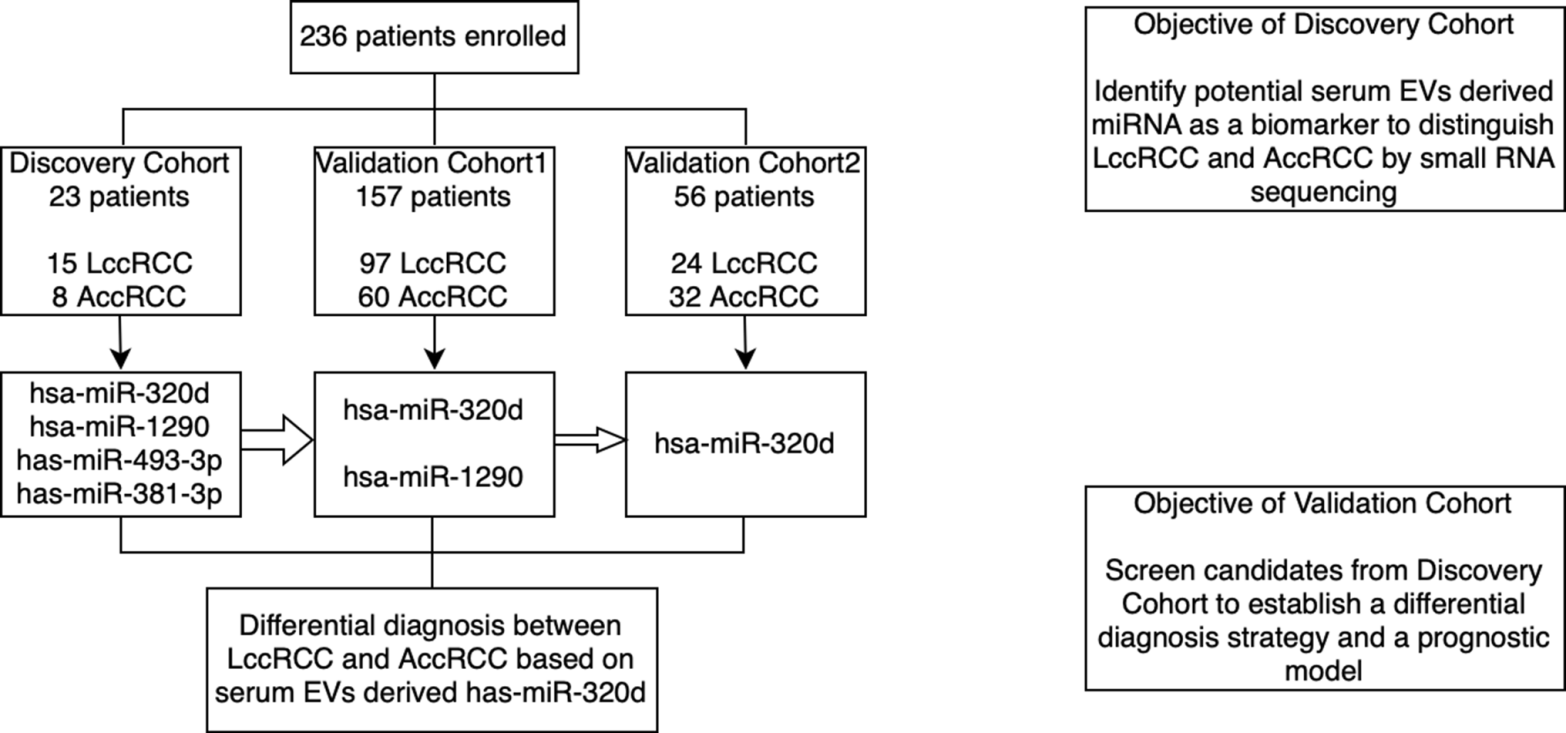
Design of the study

In order to ascertain a liquid biomarker for ccRCC prognosis, our study focused on serum derived EVs. Extracted EVs were validated using NTA (Fig. S1A), western blotting (Fig. S1B) and TEM (Fig. S1C). Our research recruited 236 patients diagnosed with ccRCC who underwent surgery at Renji Hospital from 2017.01 to 2020.12 (Fig. 1). We identified potential biomarkers in the discovery phase through small RNA sequencing and subsequently validated these candidates in two seperate phases utilizing different technologies.

### Small RNA sequencing identified serum EVs derived miRNAs as prognostic biomarker of ccRCC

**Fig. 2:**
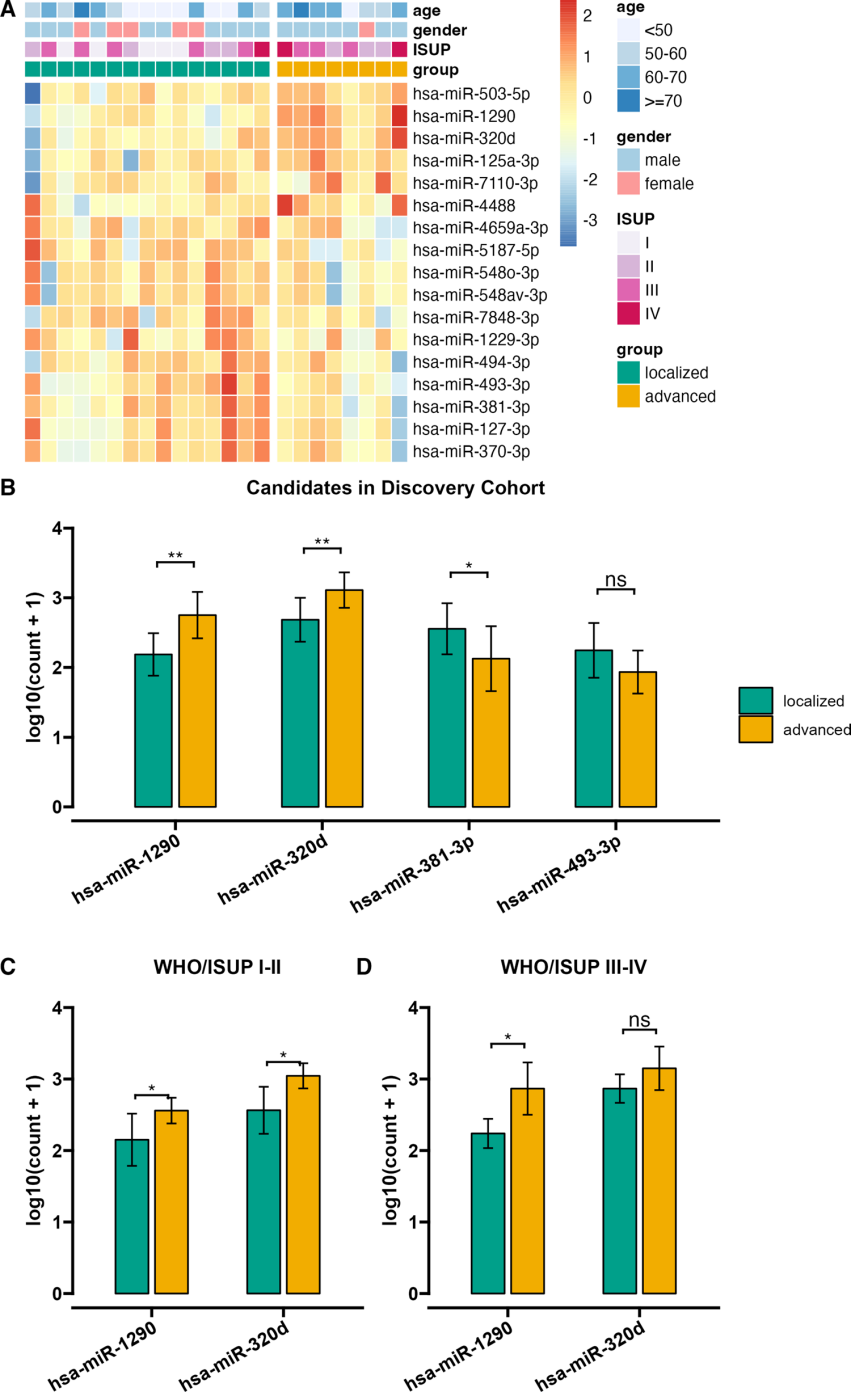
Discovery of differentially expressed EVs derived miRNAs in LccRCC and AccRCC. **A** Heatmap of high throughput small RNA sequencing. **B** Candidates in discovery cohort. **C** Candidates in the subgroup WHO/ISUP I-II. D)Candidates in the subgroup WHO/ISUP III-IV. ns, no significance. *, p < 0.05. **, p < 0.01. ***, p < 0.001

Fifteen patients with localized ccRCC(LccRCC)(T1-2N0M0) and eight patients with advanced ccRCC(AccRCC)(T3-4N0-1M0-1, including recurrent ccRCC, ccRCC with vena thrombus, ccRCC with fat invasion, ccRCC with lymph node invasion and metastatic ccRCC) were enrolled for miRNA screening [25] (Table 1).Total RNA was extracted from serum EVs, and a profiled small RNA deep sequencing assay was performed. Differential expression of miRNAs was compared between LccRCC and AccRCC (Fig. 2A, B), as well as in the WHO/ISUP subgroups (Fig. 2C, D). This screening identified two potential miRNA candidates, hsa-miR-320d and hsa-miR-1290, which were identified as significantly upregulated in AccRCC versus LccRCC.

**Table 1:** Clinical information of the discovery cohort

ID	Group	WHO/ ISUP	Pathological Stage	SEX	AGE	SIDE	Pathological Diagnosis
1	LccRCC	I	I	MALE	51	Left	ccRCC
2	LccRCC	I	I	FEMALE	41	Right	ccRCC
3	LccRCC	I	I	MALE	43	Left	ccRCC
4	LccRCC	I	I	MALE	66	Left	ccRCC
5	LccRCC	I	I	MALE	46	Right	ccRCC
6	LccRCC	II	I	MALE	56	Right	ccRCC with degeneration and hemorrhage
7	LccRCC	II	I	FEMALE	46	Left	ccRCC
8	LccRCC	II	I	MALE	44	Right	ccRCC
9	LccRCC	II	I	MALE	36	Right	ccRCC
10	LccRCC	III	I	FEMALE	72	Tight	ccRCC
11	LccRCC	III	I	MALE	63	Right	ccRCC with hemorrhage
12	LccRCC	III	I	FEMALE	53	Right	ccRCC with necrosis
13	LccRCC	III	I	FEMALE	67	Left	ccRCC
14	LccRCC	III	I	MALE	69	Left	ccRCC
15	LccRCC	IV	I	MALE	59	Left	ccRCC with sacromatoid
16	AccRCC	II	IV	MALE	55	Right	recurrent ccRCC
17	AccRCC	I-II	IV	FEMALE	57	Right	ccRCC with lung metastasis
18	AccRCC	II	IV	MALE	69	Right	ccRCC with thrombus, adrenal invasion and paravena cava lymph nodes metastasis
19	AccRCC	III	IV	MALE	39	Left	recurrent ccRCC with necrosis
20	AccRCC	III	IV	MALE	73	Left	recurrent ccRCC with necrosis
21	AccRCC	III	IV	MALE	67	Right	ccRCC with bone metastasis
22	AccRCC	IV	IV	MALE	68	Left	ccRCC with thrombus,renal sinus fat invasion,and para-aortic lymph nodes in hilum metastasis
23	AccRCC	III-IV	IV	MALE	68	Right	ccRCC with degeneration, necrosis, and lymphocytes infiltration

### Validation of serum EVs derived hsa-miR-320d as a prognostic biomarker distinguishing different stages of ccRCC

**Fig. 3:**
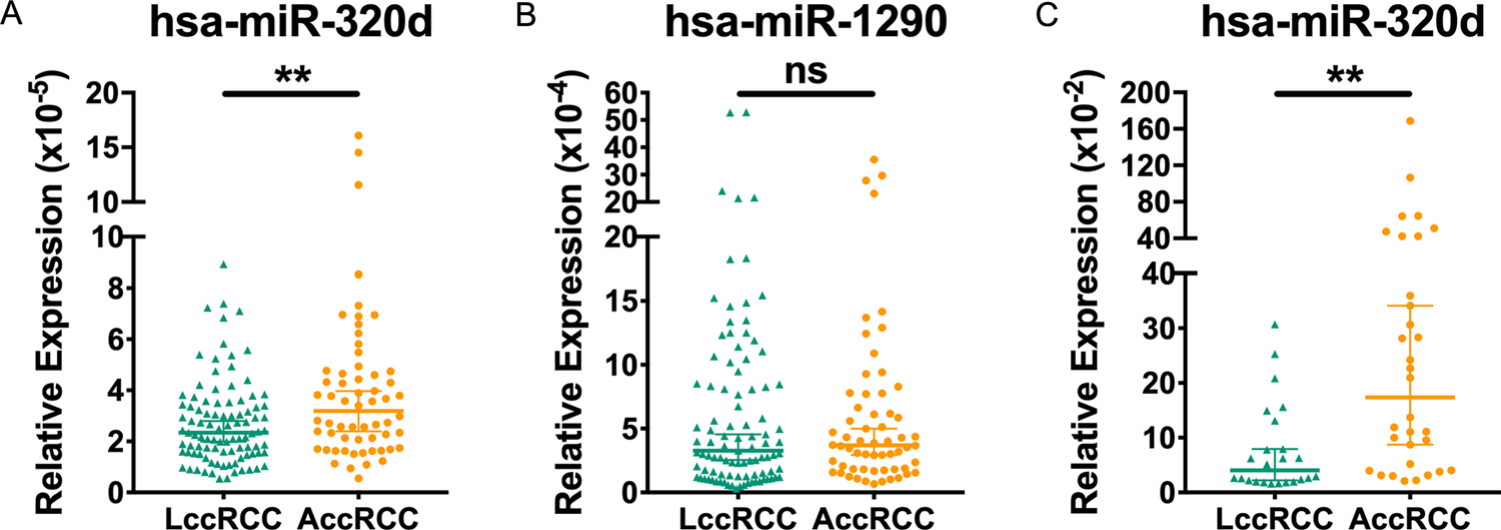
Validation of candidate EVs derived miRNAs in two independent cohorts. **A** Relative expression to cel-miR-40 of hsa-miR-320d of LccRCC and AccRCC in cohort 1. **B** Relative expression to cel-miR-40 of hsa-miR-1290 of LccRCC and AccRCC in cohort 1. **C** Relative expression to cel-miR-39 of hsa-miR-320d of LccRCC and AccRCC in cohort 2. ns, no significance. **, p < 0.01

Due to the difficulty in detecting hsa-miR-493-3p, a down-regulated miRNA (Figure S2), our focus shifted to the up-regulated hsa-miR-320d and hsa-miR-1290 in order to validate their prognostic value. To accomplish this, we established a validation cohort comprising 97 LccRCCs and 60 AccRCCs (Table 2). Exogenous cel-miR-40-3p was introduced to serum EVs derived total RNA for normalization. The expression levels of hsa-miR-320d and hsa-miR-1290 were compared between the two groups, revealing that only the over expression of hsa-miR-320d was able to distinguish AccRCC from LccRCC (p < 0.01), while expression of hsa-miR-1290 did not exhibit a significant difference (Fig. 3A, B and Fig. S2A). In another validation cohort comprising of 24 LccRCC and 32 AccRCC with exogenous cel-miR-39 added as normalization (Table 2, Fig. 3C), the findings were consistent with the previous result, indicating that the expression of serum EVs derived hsa-miR-320d was significantly upregulated in AccRCC.

**Table 2:** Clinical information of two independent validation cohorts

Parameters	LccRCC (n = 97) (%)	AccRCC (n = 60) (%)
Sex		
Male	65 (67)	46 (77)
Female	32 (33)	14 (23)
Age		
20–45	20 (21)	5 (8)
46–59	32 (33)	24 (40)
60–69	33 (34)	21 (35)
70–85	12 (12)	10 (17)
WHO/ISUP		
I	21 (22)	3 (5)
II	55 (56)	20 (33)
III	21 (22)	27 (45)
IV	0 (0)	10 (17)
Maximum diameter (cm)		
0–4	58 (60)	11 (18)
4–7	36 (37)	26 (43)
7–10	2 (2)	15 (25)
10–15	1 (1)	8 (14)
Necrosis		
Yes	1 (1)	23 (38)
No	96 (99)	37 (62)

Parameters	LccRCC (n = 24) (%)	AccRCC (n = 32) (%)
Sex		
Male	20 (83)	28 (88)
Female	4 (17)	4 (12)
Age		
20–45	0 (0)	3 (9)
46–59	9 (38)	6 (19)
60–69	12 (50)	15 (47)
70–85	3 (12)	8 (25)
WHO/ISUP		
I	4 (17)	1 (3)
II	10 (42)	13 (41)
III	7 (29)	14 (44)
IV	3 (12)	4 (12)
Maximum diameter (cm)		
0–4	7 (29)	7 (22)
4–7	13 (54)	15 (47)
7–10	4 (17)	6 (19)
10–15	0 (0)	4 (12)
Necrosis		
Yes	3 (12)	8 (25)
No	21 (88)	24 (75)

### Validation of serum EVs derived hsa-miR-320d as a prognostic biomarker predicting metastasis and recurrence of ccRCC

**Fig. 4:**
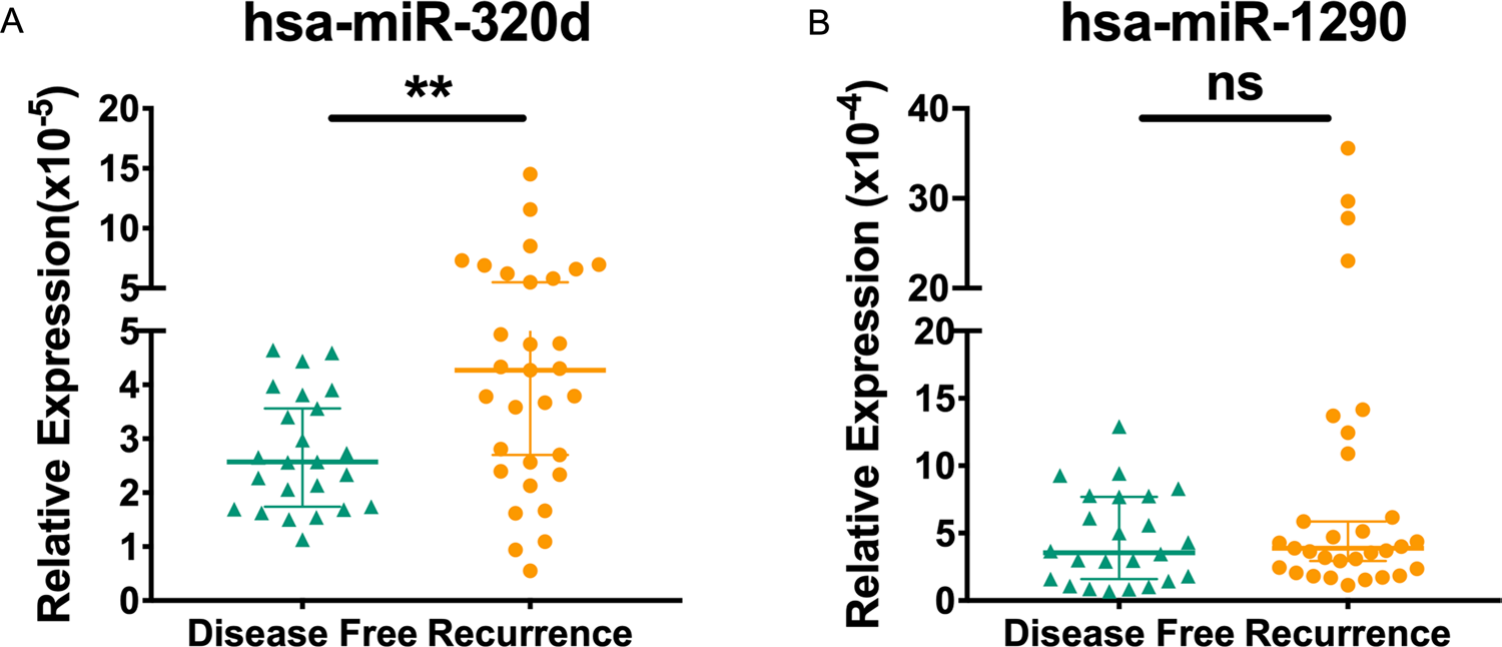
EVs derived hsa-miR-320d predicts early relapse of AccRCC. **A** Relative expression to cel-miR-40 of hsa-miR-320d distinguish disease free or recurrence/metastasis in AccRCC in cohort 1. **B** Relative expression to cel-miR-40 of hsa-miR-1290 distinguish disease free or recurrence/metastasis in AccRCC in cohort 1. ns, no significance. **, p < 0.01

To further investigate the prognostic significance, we conducted a matching analysis of the post-surgery follow-up information with the expression levels. After excluding the five missing patients 51.7% patients with AccRCC experienced recurrence or metastasis, while 40% remained disease free within two years after surgery (Table 3). As few LccRCC patients exhibited metastasis or recurrence, we found that the serum EVs derived hsa-miR-320d was significantly upregulated in patients with progressed AccRCC (p < 0.01), while the expression of hsa-miR-1290 showed no significant difference between these two groups (Fig. 4A, B). Interestingly, according to the starBase v3.0 project, although hsa-miR-320d is upregulated in tumor tissue (Fig. S3A), its expression level in tumor tissue has no prognostic value (Fig. S3B).

**Table 3:** Clinical and follow-up information of the AccRCC subgroup in validation cohort1

Parameters	Disease free (n = 24) (%)	Recurrence (n = 31) (%)	Missing (n = 5) (%)
Sex			
Male	17 (71)	26 (84)	3 (60)
Female	7 (29)	5 (16)	2 (40)
Age			
20–45	2 (9)	2 (6)	1 (20)
46–59	8 (33)	13 (42)	3 (60)
60–69	8 (33)	12 (39)	1 (20)
70–85	6 (25)	4 (13)	0 (0)
WHO/ISUP			
I	1 (4)	0 (0)	2 (40)
II	11 (46)	8 (26)	1 (20)
III	10 (42)	16 (52)	1 (20)
IV	2 (8)	7 (22)	1 (20)
Maximum diameter (cm)			
0–4	6 (25)	5 (16)	0 (0)
4–7	8 (34)	14 (45)	4 (80)
7–10	6 (25)	9 (29)	0 (0)
10–15	4 (16)	3 (10)	1 (20)
Necrosis			
Yes	5 (21)	18 (58)	0 (0)
No	19 (79)	13 (42)	5 (100)

### Hsa-miR-320d enhances the pro-metastatic phenotype of ccRCC cells in vitro

**Fig. 5:**
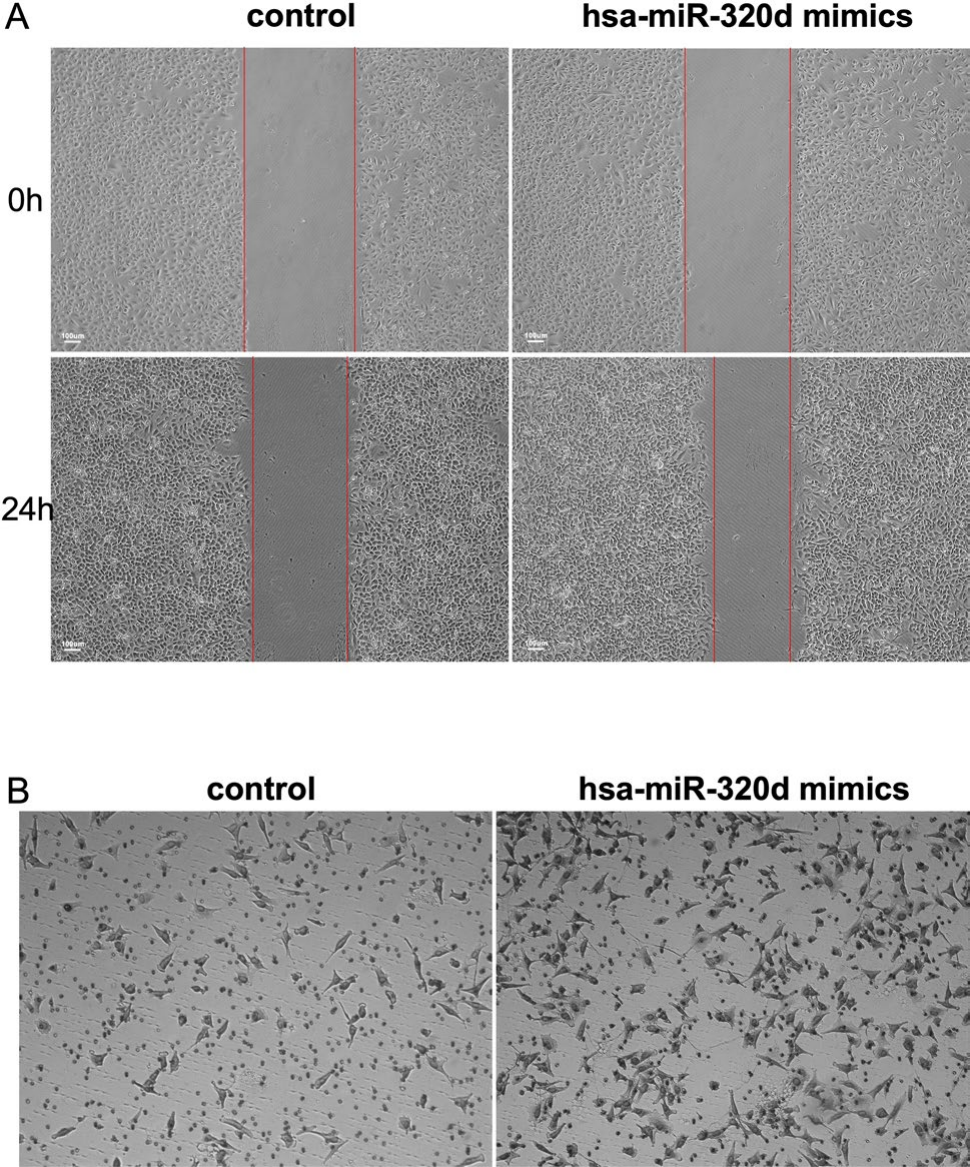
Hsa-miR-320d promotes OSRC2 migration and invasion. **A** Cell migration ability of OSRC2 transfected with hsa-miR-320d was assessed by wound healing assay. **B** Cell migration ability of OSRC2 transfected with hsa-miR-320d was assessed by matrigel invasion assay

In order to gain a deeper understanding of the relationship between the upregulation of hsa-miR-320d indicates ccRCC metastasis, a transfection of hsa-miR-320d mimic was performed on the ccRCC cell line OSRC2. Wound healing assays demonstrated that the overexpression of hsa-miR-320d significantly promoted cell migration in OSRC2 (Fig. 5A). Additionally, transwell assays also proved that the invasion of OSRC2 was enhanced following exogenous hsa-miR-320d stimulation (Fig. 5B). Besides, the overexpression of hsa-miR-320d did not influence the proliferation of OSRC2 (Fig. S4A).

## Discussion

Our study utilized small RNA sequencing to identify novel candidates, which were subsequently validated in a sizable cohort with a minimum follow-up period of two years. Obvious differences were observed between patients with favorable prognosis or unfavorable prognosis with serum EVs derived hsa-miR-320d emerging as the most promising candidate. To ensure the reliability of our findings, two distinct methods were employed to extract EVs derived total RNA and two acknowledged external controls were used. Our results provide compelling evidence that hsa-miR-320d is highly expressed in serum derived EVs in advanced ccRCC and may serve as a ccRCC progression promoter. This is consistent with the TCGA public database that hsa-miR-320d is upregulated in various cancers including ccRCC. [26, 27]

Because of the relatively favorable prognosis of early stage ccRCC after surgery, metastasis and recurrence contributes the most mortality of ccRCC [28]. However, monitoring ccRCC poses a challenge due to the absence of suitable biomarkers. [29]. CT or MR is time consuming and uneconomical, and metastasis is seldom limited to the renal recess [30]. Liquid biopsy, based on miRNA, circulating tumor cells(CTCs), circulating tumor DNA(ctDNA), or EVs can provide clue and move the detection of recurrence or metastasis ahead [31]. In the context of ccRCC, CTCs are difficult to capture because of the negatively EpCAM expression in most RCC cells [32]. In addition, unlike prostate cancer, mutations in ctDNA are incompatible with mutations in parent tumors, and it is difficult to provide information on the tumor load [33]. Given their membrane structure and stability, extracellular vesicles (EVs) hold promise as an optimal candidate for liquid biopsy in ccRCC.

Since EVs facilitate the intercellular transmission of molecules, they play an important role in cancer development [11]. EVs can promote cancer proliferation and invasion by delivering metabolites, nucleic acids, or proteins, thus EVs have been identified as biomarkers of several types of cancer, including colorectal cancer [34, 35], gastric cancer [36], and prostate cancer [37]. In addition, taking advantage of nano materials, bioengineered EVs have been applied in drug delivery [38], showing the great potential of EVs [39].

Several previous studies have focused on the role of EVs in RCC. EVs are involved in the progression of RCC and participate in angiogenesis [40, 41], invasion and metastasis [19, 42] and immune regulation [43], with different origins and through distinct pathways. However, there exists a disparity between these cell line-based studies and the potential biomarkers. Our research focused on taken EVs derived miRNA as a biomarker of ccRCC, which is somehow similar to previous studies that focused on functionally well-researched miRNAs, such as serum EVs derived miR-210 [21], plasma EVs derived miR-615-3p [23], or urine EVs derived miR-126-3p [44]. To identify a novel EVs derived miRNA, our study took advantage of small RNA sequencing and found that hsa-miR-320d, hsa-miR-1290 which have not been previously researched in ccRCC, can potentially become biomarkers of ccRCC and were finally validated in more than 200 patients.

In vitro experiments also supported that hsa-miR-320d improved the metastatic potency of ccRCC cells, which is in concordance with our conclusion that EVs derived hsa-miR-320d is a biomarker of ccRCC progression. We hypothesized that miR-320-d might down-regulate PPP2CA expression, resulting in increased phosphorylation of AKT, which further induced nuclear translocation of β-catenin/NF-κB to activate transcription of EMT-related genes [45, 46] (Fig. S4B).

Our study was subject to several limitations, mainly due to it is a retrospective single-center analysis. Firstly, widely used prognostic models such as UISS or Leibovich have demonstrated significant potential in predicting the metastasis or recurrence of ccRCC. However, as a liquid biomarker, serum EVs derived from hsa-miR-320d are independent of pathological findings, thereby providing an additional dimension for prognostic assessment. Secondly, the recruitment of only surgically treated patients may have introduced selection bias. For example, most patients in the LccRCC cohort exhibited a pathologic stage T1a, indicating a highly favorable prognosis, which restricts the application of EVs derived hsa-miR-320d. Furthermore, the absence of widespread use of immune therapies and TKIs within our cohort precludes the evaluation of the potential of EVs derived hsa-miR-320d as a biomarker for treatment response. Additionally, the acquisition of serum samples was constrained, thereby impeding the comparison between pre-treatment and post-treatment data.

In conclusion, the up-regulation of serum EVs derived hsa-miR-320d in ccRCC patients with an unfavorable prognosis, and its ability to enhance the pro-metastatic phenotype of ccRCC cells suggest that serum EVs derived hsa-miR-320d as a liquid biomarker has a great potential for detecting the recurrence or metastasis of ccRCC.

## Supplementary Information

Supplementary file1 (JPG 553KB)

Supplementary file2 (PDF 69KB)

Supplementary file3 (JPG 1220KB)

Supplementary file4 (JPG 244KB)

## Data Availability

Please contact author for data requests.

## Abbreviations

EVs: Extracellular vesicles
RCC: Renal cell carcinoma
ccRCC: Clear cell renal cell carcinoma
LccRCC: Localized clear cell renal cell carcinoma
AccRCC: Advanced clear cell renal cell carcinoma
