# Supplementary figures and images for: Serum extracellular vesicles derived hsa-miR-320d as an indicator for progression of clear cell renal cell carcinoma
Source: Discov Oncol. 2023 Jun 28;14:114. doi: 10.1007/s12672-023-00730-2 (PMC10307730; doi:10.1007/s12672-023-00730-2)

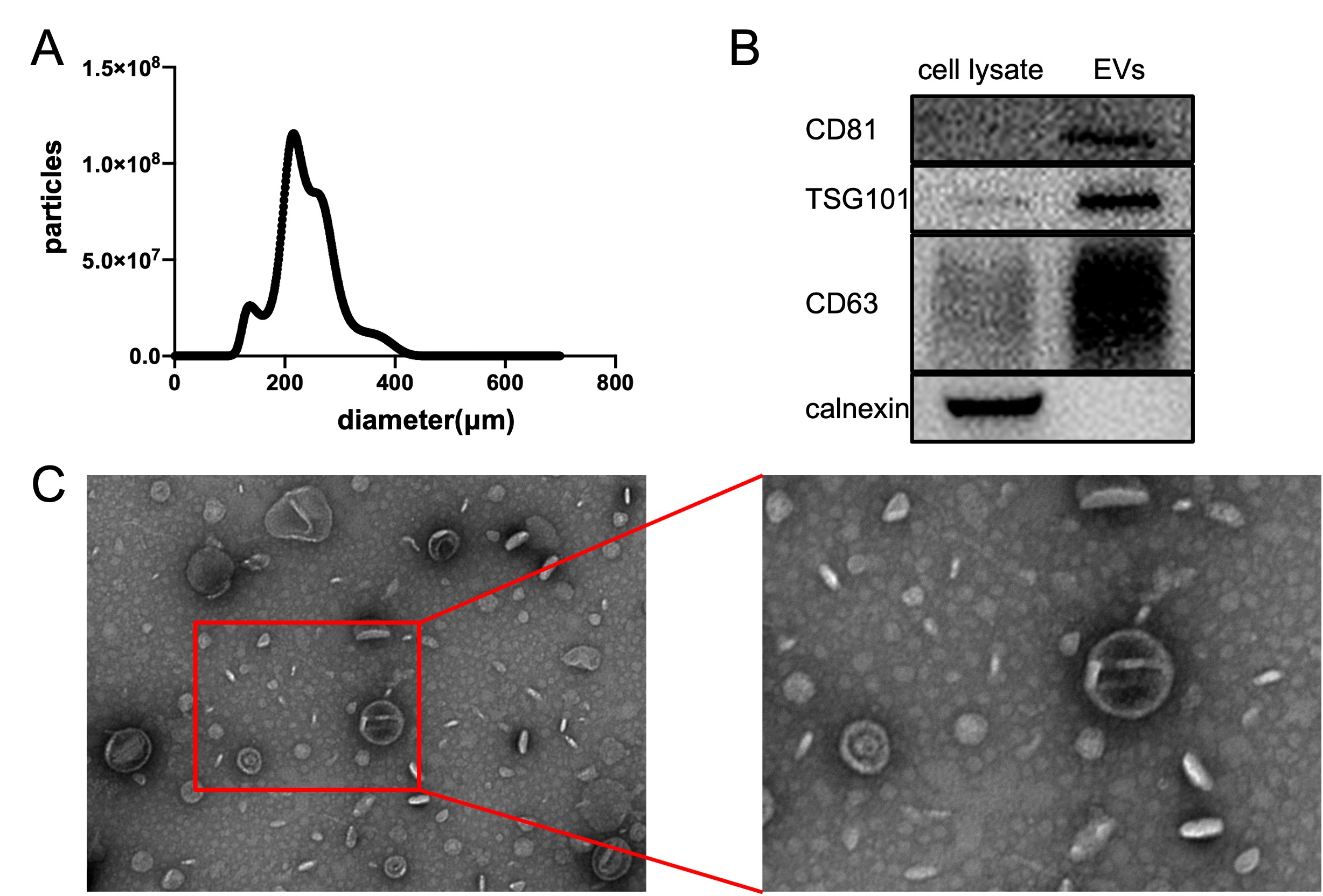

Supplement: Supplementary file 1 — Supplementary file1 (JPG 553KB) [file 12672_2023_730_MOESM1_ESM.jpg]

A

hsa-miR-1290

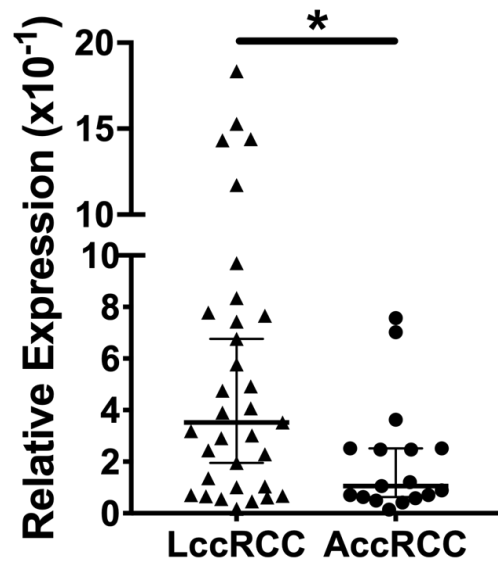

B

hsa-miR-493-3p

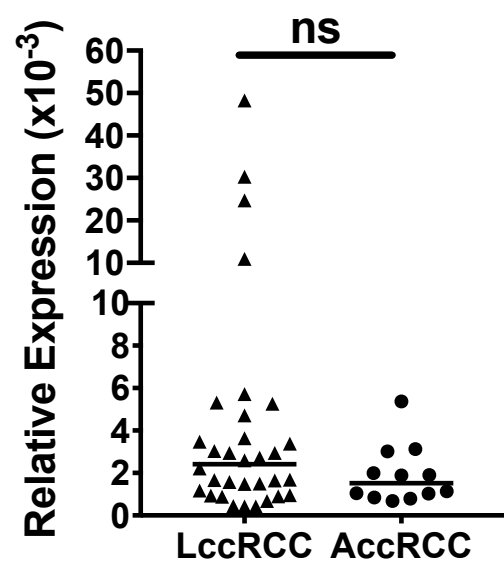

Supplement: Supplementary file 2 — Supplementary file2 (PDF 69KB) [file 12672_2023_730_MOESM2_ESM.pdf]

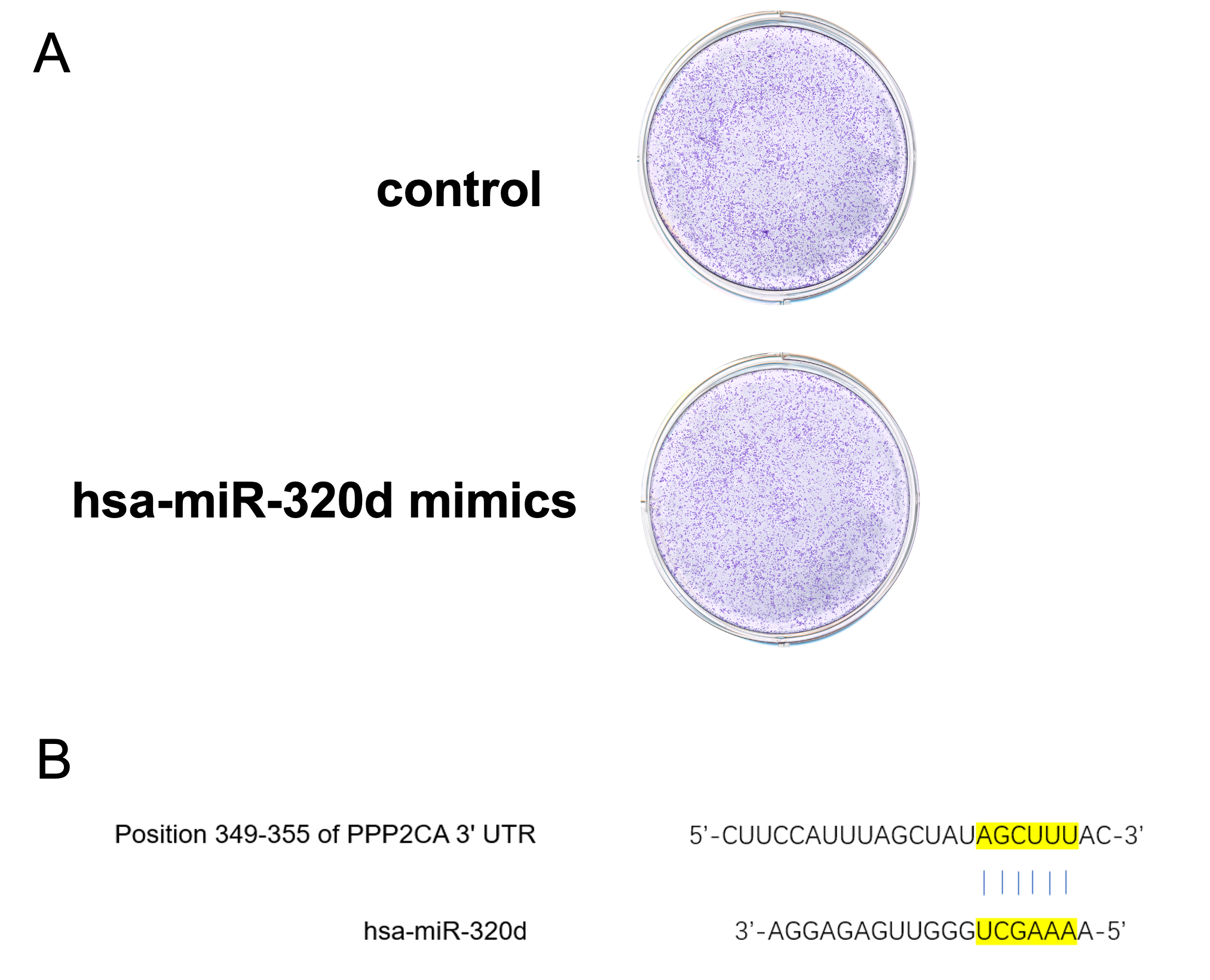

Supplement: Supplementary file 3 — Supplementary file3 (JPG 1220KB) [file 12672_2023_730_MOESM3_ESM.jpg]

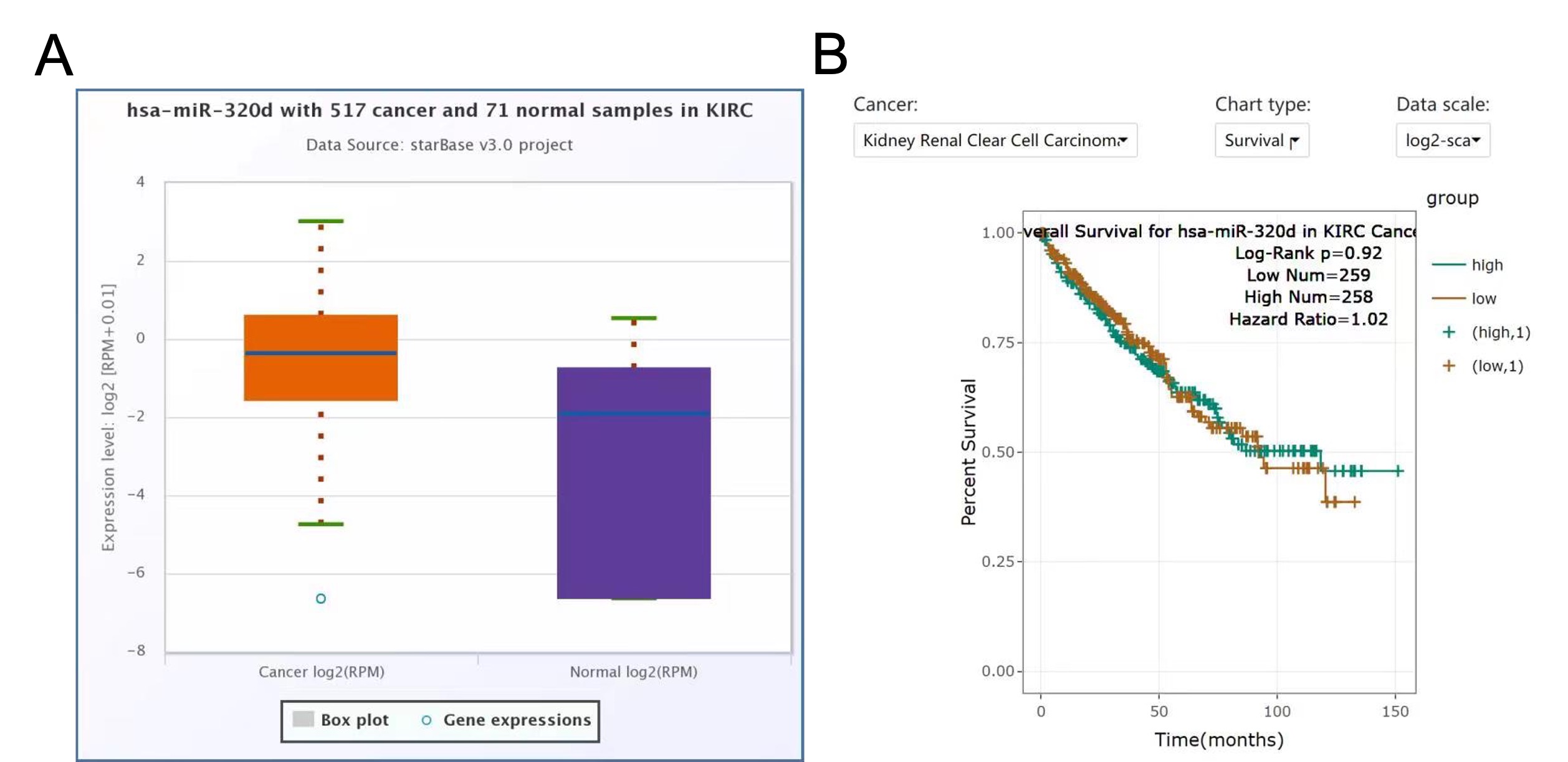

Supplement: Supplementary file 4 — Supplementary file4 (JPG 244KB) [file 12672_2023_730_MOESM4_ESM.jpg]
